# Reference DNA barcodes and other mitochondrial markers for identifying Caribbean Octocorals

**DOI:** 10.3897/BDJ.7.e30970

**Published:** 2019-02-20

**Authors:** Jaime G. Morín, Dagoberto E. Venera-Pontón, Amy C. Driskell, Juan A. Sánchez, Howard R. Lasker, Rachel Collin

**Affiliations:** 1 Laboratorio de Sistemática Molecular y Filogeografía, Facultad de Ciencias Biológicas, Universidad Nacional Mayor de San Marcos, Lima, Peru Laboratorio de Sistemática Molecular y Filogeografía, Facultad de Ciencias Biológicas, Universidad Nacional Mayor de San Marcos Lima Peru; 2 Smithsonian Tropical Research Institute, Panama City, Panama Smithsonian Tropical Research Institute Panama City Panama; 3 Laboratories of Analytical Biology, National Museum of Natural History, Smithsonian Institution, Washington, D.C., United States of America Laboratories of Analytical Biology, National Museum of Natural History, Smithsonian Institution Washington, D.C. United States of America; 4 Laboratorio de Biología Molecular Marina – BIOMMAR, Bogotá, Colombia Laboratorio de Biología Molecular Marina – BIOMMAR Bogotá Colombia; 5 Department of Geology, University at Buffalo, Buffalo, United States of America Department of Geology, University at Buffalo Buffalo United States of America; 6 Smithsonian Tropical Research Institute, Balboa, Panama Smithsonian Tropical Research Institute Balboa Panama

**Keywords:** cytochrome oxidase I, 16S, Panama, Bocas del Toro, gorgonian, DNA barcoding

## Abstract

DNA barcoding is a useful tool for documenting the diversity of metazoans. The most commonly used barcode markers, 16S and COI, are not considered suitable for species identification within some "basal" phyla of metazoans. Nevertheless metabarcoding studies of bulk mixed samples commonly use these markers and may obtain sequences for "basal" phyla. We sequenced mitochondrial DNA fragments of cytochrome oxidase c subunit I (COI), 16S ribosomal RNA (16S), NADH dehydrogenase subunits 2 (16S-ND2), 6 (ND6-ND3) and 4L (ND4L-MSH) for 27 species of Caribbean octocorals to create a reference barcode dataset and to compare the utility of COI and 16S to other markers more typically used for octocorals. The most common genera (*Erythropodium*, *Ellisella*, *Briareum*, *Plexaurella*, *Muriceopsis* and *Pterogorgia*) were effectively distinguished by small differences (5 or more substitutions or indels) in COI and 16S sequences. *Gorgonia* and *Antillogorgia* were effectively distinguished from each other by unique haplotypes, but the small genetic differences make distance approaches ineffective for these taxa. *Plexaura, Pseudoplexaura* and *Eunicea* were indistinguishable from each other but were generally effectively distinguished from other genera, further supporting the idea that these genera have undergone a rapid endemic radiation in the Caribbean.

## Introduction

DNA barcoding is a useful tool for documenting the diversity of most metazoan groups (Bucklin et al. 2011). Short, easily amplifiable fragments that vary amongst closely related species are sequenced from specimens identified by experts and then used as a tool to identify sequences from unidentified samples (Hebert and Gregory 2005, Bucklin et al. 2011, Hajibabaei et al. 2007). This approach is useful in a variety of contexts (Bucklin et al. 2011, Bucklin et al. 2016, Fonseca et al. 2010, Hajibabaei et al. 2007); however the most commonly used barcode marker, a fragment of cytochrome c oxidase subunit I (COI hereafter), is not suitable for species identification within some metazoan groups such as Porifera and Anthozoa (Shearer et al. 2002, Huang et al. 2008). Consequently, less effort has been invested in building reference databases of COI and 16S barcodes within these taxa, as opposed to those groups where these markers are perceived as useful for species identification.

DNA barcoding has been used extensively to detect cryptic diversity within clades, with specific studies generally focusing on diversity within a single family, class or order. When COI is problematic, alternate barcodes are used, for example, 16S is commonly used as the molecular barcode in hydrozoans ([Bibr B4708654][Bibr B4708907]). For some clades, significant effort has been spent on identifying reliable, divergent markers and building extensive databases of these sequences for identification purposes (e.g. van der Ham et al. 2009, McFadden et al. 2014).

Recent assessments of biodiversity are increasingly focusing on metabarcoding analyses of bulk mixed samples, such as gut contents (Leray et al. 2013, Oliverio et al. 2009), settlement plates (Zaiko et al. 2016), plankton samples (Bucklin et al. 2016), sediment samples (Fonseca et al. 2010) or environmental DNA from water samples (Stat et al. 2017, Thomsen et al. 2012). These samples are typically analysed using markers that amplify across the most diverse set of taxa; most commonly COI and sometimes 16S for studies of species diversity or the nuclear SSU 18S gene in studies aimed at higher taxonomic levels. Due to the non-selective nature of metabarcoding, this approach generates sequences for taxa where the marker is not useful for species-level identification as well as for those where it is useful. Therefore, a reference set of COI and 16S sequences for these taxa could at least provide the lowest possible identification when these problematic taxa occur in mixed samples.

Most Caribbean gorgonian octocorals are endemic and closely related (Sánchez et al. 2003, Sánchez 2016, Wirshing et al. 2005). This results in many taxa with morphologically similar sister species making it extremely difficult to differentiate a number of the species with morphological characters (Sánchez and Wishing 2005). In addition, octocorals are notoriously difficult to distinguish with molecular data (France and Hoover 2002, McFadden et al. 2010, McFadden et al. 2014, McFadden et al. 2004, van der Ham et al. 2009) since their mitochondria have a unique DNA repair mechanism that is thought to be responsible for the slow evolution of mitochondrial genes (Bilewitch and Degnan 2011). Nevertheless, gorgonians are an abundant and diverse part of the macro-fauna of Caribbean reefs, with as many as 40 species occurring on a single Caribbean reef (Lasker and Coffroth 1983, Sánchez et al. 1998, Sánchez et al. 1997, Etnoyer et al. 2010, Tsounis et al. 2018). In some places, they contribute significantly to three dimensional reef structures and provide microhabitats for macro-invertebrates and fishes (Sánchez 2016). Furthermore, octocoral abundances have increased at some locations in contrast to the overall decline in scleractinians (Edmunds and Lasker 2016, Ruzicka et al. 2013). Therefore, tools that facilitate their inclusion in biodiversity assays would be a useful addition to researchers’ toolkits.

Here we had two main objectives: (1) to generate a reference set of DNA barcode sequences for common octocorals from the Southern Caribbean and (2) determine how the 16S and COI barcode fragments compare with other mitochondrial markers in their ability to distinguish genera and, in some cases, species in the fauna of Bocas del Toro, Panama.

## Materials and Methods

A total of 180 octocoral tissue samples were collected by SCUBA-diving during the summer of 2007 from the shallow waters around the Bocas del Toro Archipelago on the Caribbean coast of Panama. Tissue samples consisted of 10-20 cm sections clipped from a distal branch for each colony. Samples were identified to species using a combination of visual identification in the field (colony morphology) and microscopic examination of spicule preparations. In some cases, individuals were intermediate in morphology or could not be identified to species and were only identified to genus. Dry tissue vouchers were deposited at the USNM and the University of Panama. Details about individual samples are provided in the BoLD project workbench (Ratnasingham and Herbert 2007).

Genomic DNA was extracted from small samples of tissue (0.5 cm^3^) from each specimen using the mouse tail kit on a Biosprint 96 (Qiagen). The resuspension volume was 200 µl. We used PCR to amplify fragments of the mitochondrial genes COI, 16S, ND6-ND3, 16S-ND2 and ND4L-MSH with the primers listed in Table 1. When the barcode (“Folmer”) fragment of COI could not be amplified with the primers derived from Folmer et al. 1994, we amplified a larger fragment that includes part of the COII gene. We then extracted the barcode sequence from this longer sequence. When neither of these amplifications was successful, we amplified a shorter COI fragment with gorgonian-specific primers. The 10 µl PCR cocktail included 0.5 µl 50 mM MgCl_2_, 0.1 µl 20 µg/µl BSA, 0.5 µl dNTPs (2.5 mM each), 0.3 µl each 10 mM primer and Biolase taq polymerase (Bioline). Annealing temperatures were 46°C for the barcode fragment of COI and ND4L-MSH, 48°C for 16S and the gorgonian-specific COI fragment and 52°C for ND6-ND3, 16S-ND2 and the long fragment of COI. Amplified PCR products were cleaned using the ExoSAP-IT protocol (Affymetrix) and sequenced using the Big Dye protocol (Thermofisher).

Sequences were screened for quality and contigs of forward and reverse sequences were produced using Sequencher 5.4.6 (Gene Codes). Only sequences with a Phred quality score of at least 30 for more than 85% of the bases were combined into contigs and used for analysis. Sequences were compared internally across our dataset and BLASTned against GenBank sequences to check for contamination or mislabelling. After this step, through an abundance of caution, all 27 ND4L-MSH sequences from a single plate were excluded from the analyses because a subset of them showed unexplained divergences from previously published GenBank sequences. No other plate of sequences in our analysis showed similar problems. Sequences of each marker were aligned with ClustalX (gap-opening penalty: 15, gap-extension penalty: 6.66, transition weight: 0.5, delay divergent cutoff: 30%) and used to generate a matrix of pairwise differences [including the number of substitutions and indels (insertions/deletions)] between all the sequenced specimens. This matrix was then used to build heatmaps displaying the average pairwise differences between species. The patterns observed in heatmaps were contrasted with those observed in maximum parsimony and neighbour-joining trees (BIONJ, Gascuel 1997) built with the same matrix of pairwise differences.

Most markers showed two groups within which the pairwise differences amongst species (and genera) were consistently small. For these groups, we constructed haplotype networks for all the markers using Haplotype Viewer (Center for Integrative Bioinformatics Vienna; http://www.cibiv.at/) to determine if haplotypes were unique or related to particular species or genera.

## Data Resources

The data underpinning the analysis reported in this paper are deposited in the Barcode of Life Database (dx.doi.org/10.5883/DS-OCTOCORA) (Ratnasingham and Herbert 2007) and the barcode markers have the following GenBank numbers COI:MK153303-MK153482 and 16S:MK153483-MK153626.

## Results and Discussion

We were able to collect and successfully sequence 28 species represented by 180 individuals of octocorals from Bocas del Toro (Table 2). This represents 45% of the 61 species and includes 12 of the sixteen genera and all seven families reported for the region (Sánchez and Wishing 2005). Most markers were sequenced successfully for most species but 16S-ND2 could not be amplified for the genus *Erythropodium*, whereas the ND4L-MSH fragment failed to amplify in 5 species. Likewise, samples of *Pterogorgia anceps* almost entirely failed to amplify. Since all the specimens were processed with the same methods, differences in sequencing success may be due to taxon-specific secondary metabolites interfering with PCR.

New additions to GenBank from this study include COI for 19 species, 16S for 20 species, 16S-ND2 and ND4L-MSH for 3 species and 16S-ND2 and ND6-ND3 for 13 species (Table 2). Of those markers that were already in GenBank for a particular species, >40 of our sequences extended significantly beyond the previously reported fragment. Our samples also increased the geographic coverage of these species. For example, the majority of 16S-ND2 and ND4L-MSH sequences already in GenBank were collected from the Bahamas (Sánchez et al. 2003), so the addition of sequences from Panama provides new references for other areas of the species ranges.

**Figure 1.**
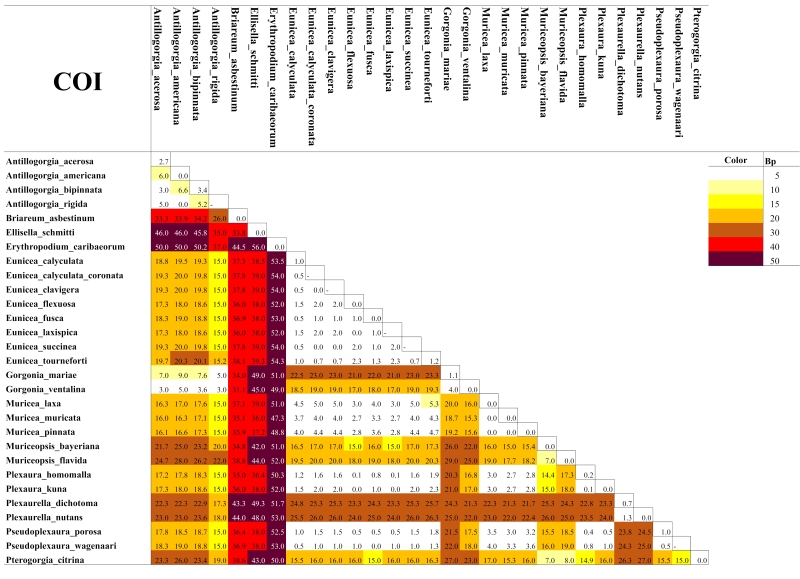
Heatmap of the mean pairwise differences between species of Caribbean octocorals, based on nucleotide substitutions in their COI sequences. Colours on the diagonal indicate the mean intra-species differences. Species with a dash in the diagonal were represented by only one individual and thus their intra-species differences could not be calculated.

**Figure 2.**
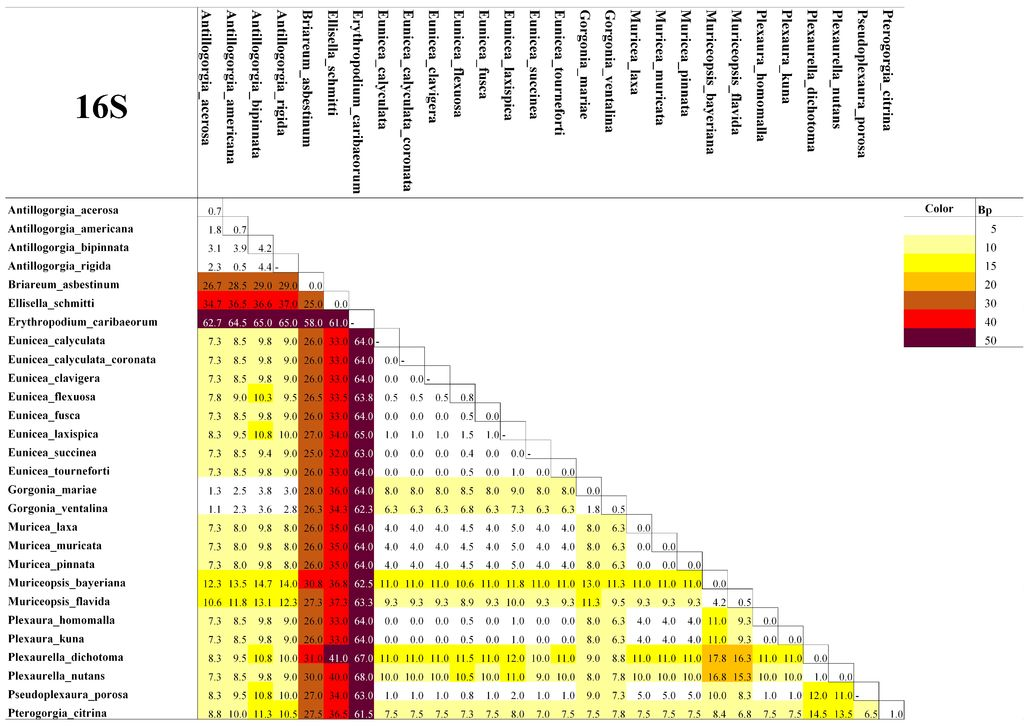
Heatmap of the mean pairwise differences between species of Caribbean octocorals, based on nucleotide (substitutions) and indel (insertions/deletions/gaps) differences of their 16S sequences. Colours on the diagonal indicate the mean intra-species differences. Species with a dash in the diagonal were represented by only one individual and thus their intra-species differences could not be calculated.

**Figure 3.**
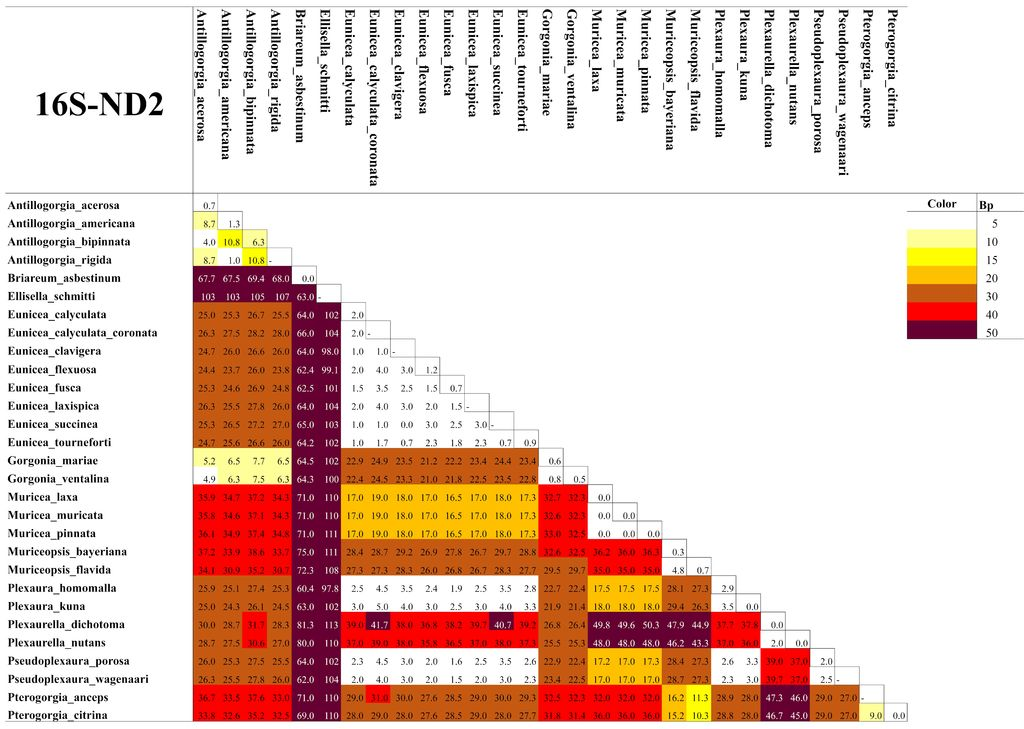
Heatmap of the mean pairwise differences between species of Caribbean octocorals, based on nucleotide (substitutions) and indel (insertions/deletions/gaps) differences of their 16S-ND2 sequences. Colours on the diagonal indicate the mean intra-species differences. Species with a dash in the diagonal were represented by only one individual and thus their intra-species differences could not be calculated.

**Figure 4.**
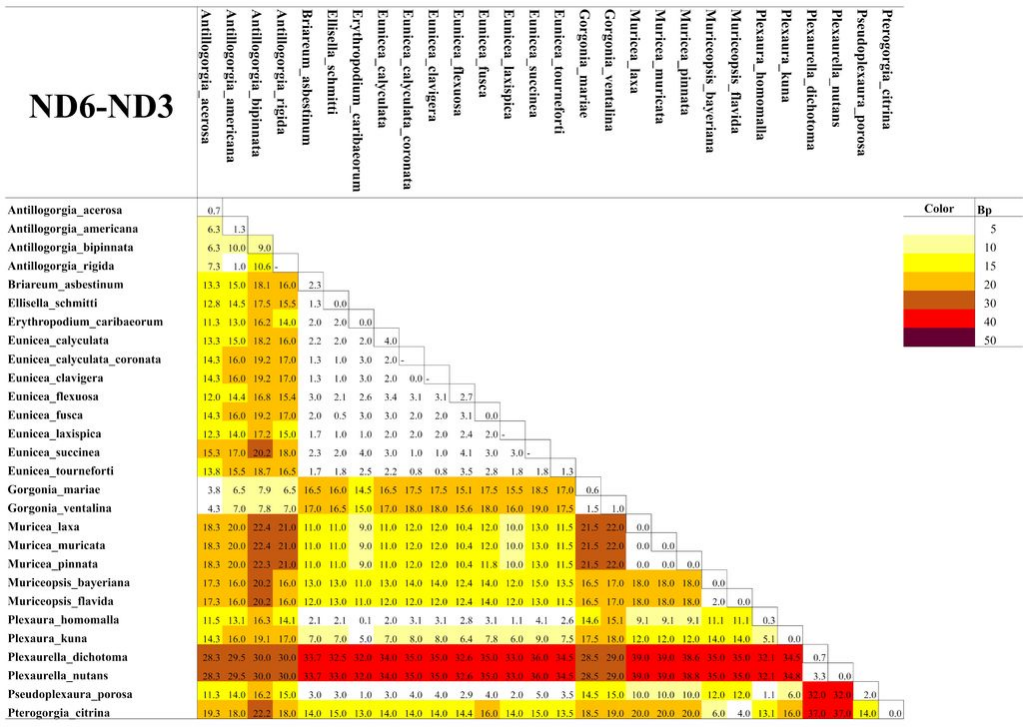
Heatmap of the mean pairwise differences between species of Caribbean octocorals, based on nucleotide (substitutions) and indel (insertions/deletions/gaps) differences of their ND6-ND3 sequences. Colours on the diagonal indicate the mean intra-species differences. Species with a dash in the diagonal were represented by only one individual and thus their intra-species differences could not be calculated.

**Figure 5.**
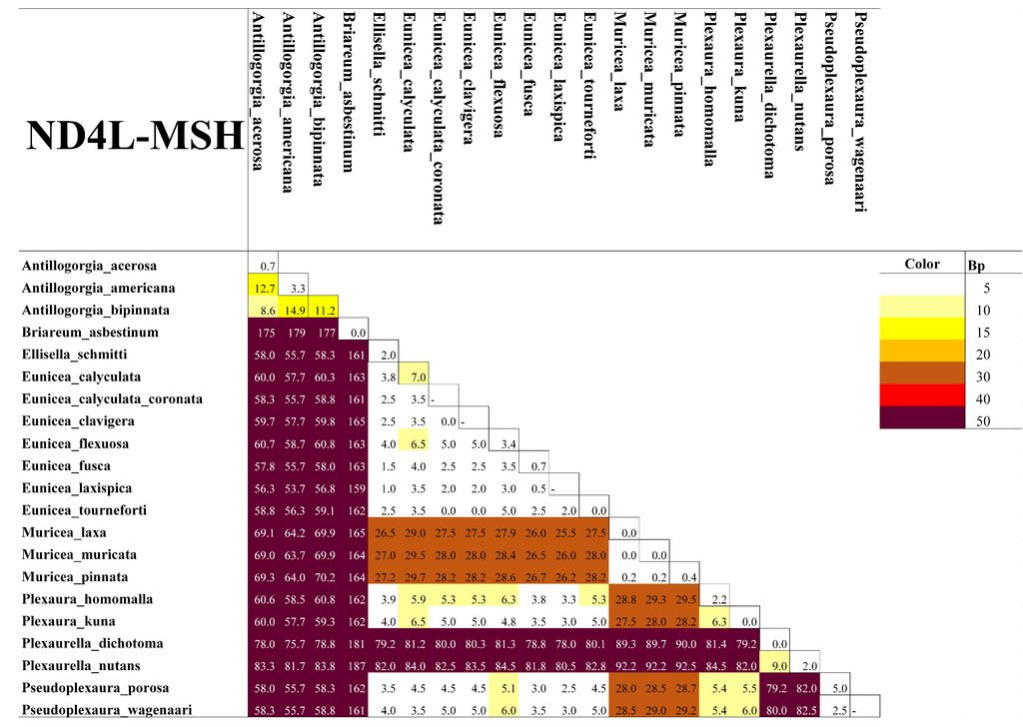
Heatmap of the mean pairwise differences between species of Caribbean octocorals, based on nucleotide (substitutions) and indel (insertions/deletions/gaps) differences of their ND4L-MSH sequences. Colours on the diagonal indicate the mean intra-species differences. Species with a dash in the diagonal were represented by only one individual and thus their intra-species differences could not be calculated.

As expected for octocorals, none of our 5 mitochondrial markers was suitable for distinguishing amongst congeneric species across the entire dataset, but they were generally useful to distinguish amongst most genera except those in the *Plexaura-Pseudoplexaura-Eunicea* group and the *Antillogorgia-Gorgonia* group (Figs 1, 2, 3, 4Fig. 5). The studied markers were able to distinguish species, but their patterns of pairwise differences varied amongst the different markers. COI, 16S-ND2 and ND4L-MSH displayed higher divergences overall than did the other two markers. The genera *Briareum, Plexaurella, Erythropodium, Muriceopsis* and *Pterogorgia* were clearly distinguishable from each other. Other taxa were partially distinguishable. For example, species of *Plexaura, Pseudoplexaura* and *Eunicea* were often indistinguishable from each other but could generally be distinguished from species in other genera across the dataset. The same situation occurred with *Antillogorgia* and *Gorgonia* which had very similar haplotypes. *Ellisella* and *Erythropodium* were clearly distinguishable with COI, 16S and 16S-ND2 but surprisingly appeared to fall within the *Plexaura-Pseudoplexaura-Eunicea* clade with ND4L-MSH (*Ellisella*) and ND6-ND3 (both). We could find no problems with our sequences or data flow which would account for this, but this result should be independently confirmed for ND4L-MSH and ND6-ND3 in these two genera.

**Figure 6.**
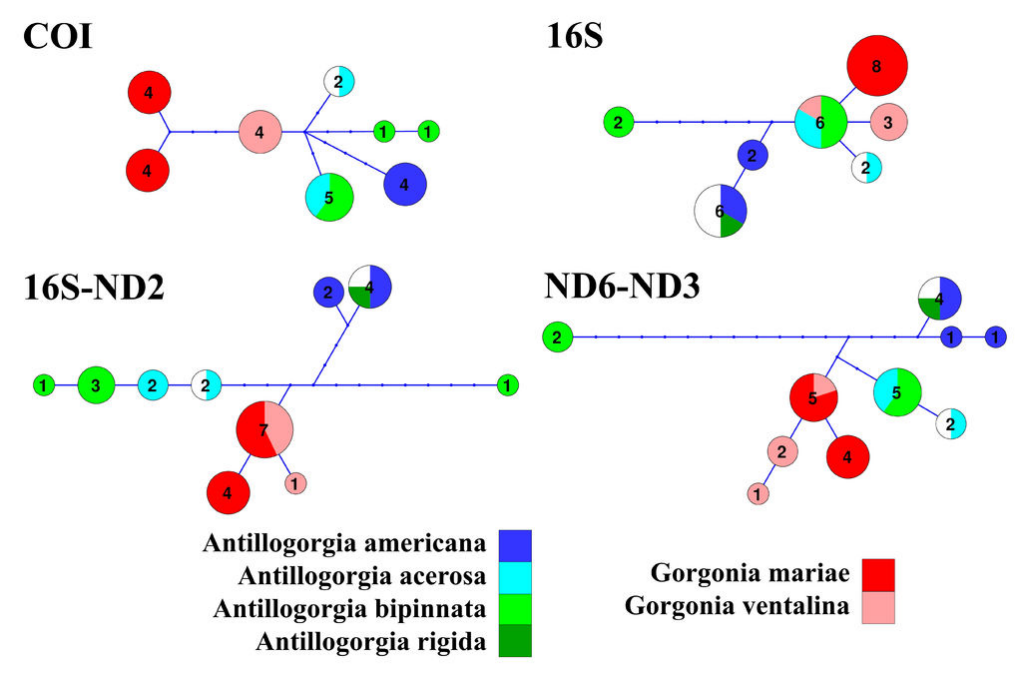
Haplotype networks involving *Gorgonia* and *Antillogorgia* DNA sequences. Each haplotype is represented by a circle. The number of individuals within each haplotype is indicated within the corresponding circle. The circle's sizes are proportional to the number of individuals. The white areas within some circles represent individuals identified only to the genus level (species unknown), whereas other colours are species-specific. The length between haplotypes represents the number of nucleotide differences between them: 1 length unit = 1 substitution or indel (insertion/deletion).

Haplotype network analyses of the *Plexaura-Pseudoplexaura-Eunicea* group and the *Antillogorgia* and *Gorgonia* group, were able to distinguish some genera and species which were not easily distinguished by distance analysis as visualised in the heatmaps. All of the markers except for 16S show that *Gorgonia* and *Antillogorgia* can be separated, as each is comprised of unique haplotypes Fig. 6, although the haplotypes generally differ by only one or a few base pairs. In addition, COI appeared to distinguish the different species within each genus. For example, the COI network clearly separated *G. mariae* from *G. ventalina* but did not clearly separate the species of *Antillogorgia* from each other. However, our small sample sizes are not a strong test of the efficacy of each marker to uniquely identify each species. Larger samples from the across the Caribbean should be sequenced before these haplotypes could be considered diagnostic.

**Figure 7.**
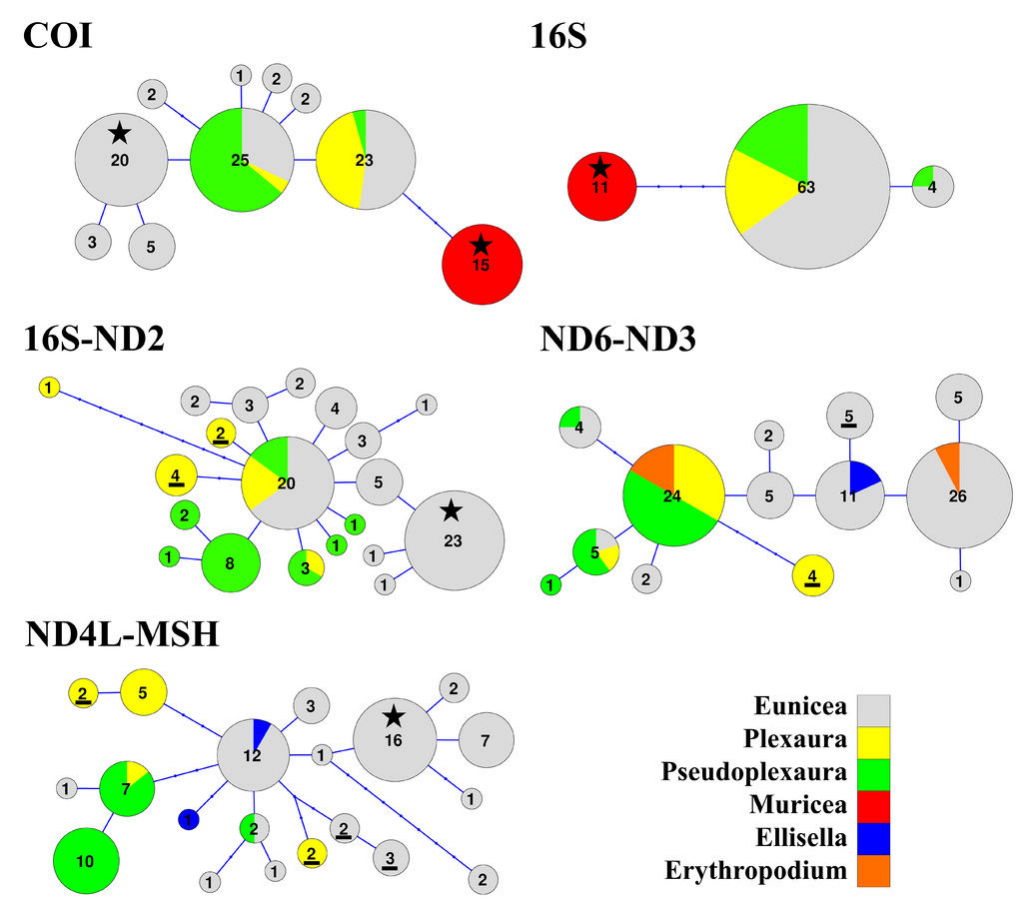
Haplotype networks of DNA sequences belonging to genera that could not be clearly separated by their nucleotide differences (heatmaps). For each gene, only the genera that were not clearly different in the heatmaps were included. Each haplotype is represented by a circle and each genus is represented by a unique colour. The number of individuals belonging to each haplotype are indicated within the corresponding circle. The circle's sizes are proportional to the number of individuals. The length between haplotypes represents the number of nucleotide differences (substitutions or indels) between them. Haplotypes present in multiple species, all belonging to the same genus, are indicated with a black star and haplotypes with all individuals belonging to the same species are indicated with underlined numbers. To be conservative, where samples only identified to genus were included in a circle, we did not consider them conspecific with samples identified to species, although they could have been.

In contrast, network analysis of the *Plexaura-Pseudoplexaura-Eunicea* group further reinforces the results of the heatmaps, indicating that these genera cannot be distinguished using any of the markers Fig. 7. For all of the makers, there were single haplotypes that were shared by *Eunicea*, *Plexaura* and *Pseudoplexaura*. *Muricea* was not clearly distinguished from the other genera using COI and 16S with the pairwise differences, but species in this genus were consistently distinguished with the network approach. These results further support the idea that the *Plexaura-Pseudoplexaura-Eunicea* group has undergone a rapid endemic radiation in the Caribbean making them particularly difficult to distinguish (Sánchez et al. 2003, Sánchez and Wishing 2005, Sánchez 2016). Likewise, both *Antillogorgia* and *Eunicea* include exemplary cases of depth-induced ecological speciation and recent divergence (Prada and Hellberg 2013, Calixto-Botía and Sánchez 2017).

As has been previously demonstrated for octocorals from other regions (Baco and Cairns 2012,France and Hoover 2002, France and Hoover 2001, McFadden et al. 2014, McFadden et al. 2004, McFadden et al. 2010), we found limited ability of the 5 markers to distinguish species based on distance methods typically employed by barcode studies Figs 1, 2, 3, 4Fig. 5. Nevertheless, metabarcoding studies are liable to obtain COI and 16S sequences from octocorals and researchers are likely to search for species identifications in BoLD or GenBank. Our sequences are the first COI and 16S sequences for many Caribbean octocoral species in GenBank and our results demonstrate that the COI barcode sequence can distinguish Caribbean species as effectively as other mitochondrial sequences more commonly used in octocoral-focused studies. Despite this, the BoLD database assigned our 28 species to only 9 Barcode Index Numbers (BINs; Ratnasingham and Hebert 2013) highlighting that alternate approaches, such as haplotype network analysis or character-based DNA barcoding (Rach et al. 2008), designed for small divergences, are more appropriate for attempts to use DNA sequences to automatically identify octocorals ([Bibr B4708664][Bibr B4978184]).

## Figures and Tables

**Table 1.** Primers used for amplification and sequencing of loci in this study and size of amplified fragments. *Lengths of fragments that include non-coding regions as well as ribosomal sequences can vary. ^1^McFadden et al. 2004; ^2^France and Hoover 2001; ^3^Meyer 2003; ^4^this study; ^5^Palumbi et al. 1991; ^6^Brugler and France 2008; ^7^Sánchez et al. 2003.

**Locus**	**Primers**	Expected Size*
**COI long**	*COII-8068F^1^* CCATAACAGGACTAGCAGCATC *COIOCTR^2^* ATCATAGCATAGACCATACC	940
**COI Folmer**	*dgLCO1490^3^* GGTCAACAAATCATAAAGAYATYGG *dgHCO2198^3^* TAAACTTCAGGGTGACCAAARAAYCA	655
**COI short**	*GorgM13_F^4^* CACGACGTTGTAAAACGACGTATGTTAGGAGATGATCATCTATAT *GorgM13_R^4^* GGATAACAATTTCACACAGGGAATGTTGTATTAAAATTYCTRTCTGT	468
**16S**	*16Sar^5^* CGCCTGTTTATCAAAAACAT *16Sbr^5^* CCGGTCTGAACTCAGATCACGT	668
**ND6-ND3**	*ND6-1487F^1^* TTTGGTTAGTTATTGCCTTT *ND3-2126R^1^* CACATTCATAGACCGACACTT	611
**16S-ND2**	*16S-647F^1^* TTTGGTTAGTTATTGCCTTT *ND2-1418R^1^* ACATCGGGAGCCCACATA	758
**ND4L-MSH**	*ND4L-2475F^6^* TAGTTTTACTGGCCTCTAC *MUT-3458R*^7^ TSGAGCAAAAGCCACTCC	870

**Table 2.** Number of individuals collected and successfully sequenced for each genetic marker for samples identified to species. The colour of the cells indicates the availability of sequences for the same marker and species in GenBank. Dark grey: A new contribution; no available sequences overlap our fragment by ≥100 bp. Light grey: Partial sequence is already available; our sequences overlap existing sequences by >350 bp; our data extends this by >50 bp. White: Sequence already available; sequences available in GenBank overlap ours completely with <50bp additional data. X mark indicates species that failed to amplify or resulted in unusable or suspicious sequences. *Sequences were also generated for an additional 4 *Antillogrogia*, 3 *Erythopodium*, 44 *Eunicea*, 3 *Muricea*, 1 *Plexaura*, 5 *Plexaurella*, 16 *Pseudoplexaura* and 9 *Pterogrogia* that were not identified to species.

**Species**	Individuals collected*	**COI**	**16S**	**16S-ND2**	**ND6-ND3**	**ND4L-MSH**
*Antillogorgia acerosa*	4	3	3	3	3	3
*Antillogorgia americana*	4	4	4	4	4	3
*Antillogorgia bipinnata*	8	5	5	5	5	4
*Antillogorgia rigida*	2	1	1	1	1	X
*Briareum asbestinum*	9	8	7	7	3	7
*Ellisella schmitti*	2	2	2	1	2	2
*Erythropodium caribaeorum*	3	3	1	X	3	X
*Eunicea calyculata*	3	3	2	3	3	3
*Eunicea clavigera*	1	1	1	1	1	1
*Eunicea flexuosa*	9	8	8	8	8	8
*Eunicea fusca*	5	4	4	4	4	4
*Eunicea laxispica*	1	1	1	1	1	1
*Eunicea succinea*	1	1	1	1	1	X
*Eunicea tourneforti*	6	6	4	6	6	3
*Gorgonia mariae*	8	8	8	8	8	X
*Gorgonia ventalina*	4	4	4	4	4	X
*Muricea laxa*	7	7	3	6	7	6
*Muricea muricata*	3	3	3	3	3	3
*Muricea pinnata*	5	5	3	5	5	5
*Muriceopsis bayeriana*	6	6	6	6	6	X
*Muriceopsis flavida*	4	3	4	3	3	X
*Plexaura homomalla*	11	8	6	8	8	7
*Plexaura kuna*	4	4	4	4	4	2
*Plexaurella dichotoma*	3	3	3	3	3	3
*Plexaurella nutans*	4	2	2	2	2	2
*Pseudoplexaura porosa*	2	2	1	2	2	2
*Pseudoplexaura wagenaari*	1	1	X	1	X	1
*Pterogorgia anceps*	4	X	X	1	X	X
*Pterogorgia citrina*	2	2	2	2	2	X
